# Impact of childbirth policy changes on obstetric workload over a 13-year period in a regional referral center in China – implications on service provision planning

**DOI:** 10.1186/s12884-021-04074-z

**Published:** 2021-09-07

**Authors:** Min Xie, Terence T Lao, Junnan Ma, Tianying Zhu, Dajin Liu, Shengnan Yu, Mingyu Du, Qian Sun, Runmei Ma

**Affiliations:** 1 Department of Obstetrics and Gynecology, the First Affiliated Hospital of Kunming Medical University, China, PO Box 650032, No.295 Xi Chang Rd, Kunming, Yunnan China; 2 Department of Obstetrics and Gynecology, the Chinese University of Hong Kong, Hong Kong, China; 3 Department of Medical Records, the First Affiliated Hospital of Kunming Medical University, Kunming, Yunnan China; 4 Kunming Angel Women and Children’s Hospital, Kunming, Yunnan China

**Keywords:** High risk pregnancy, Medical staffing, Midwifery staffing, Workload, China

## Abstract

**Background:**

We aimed to appraise the impact of the changing national childbirth policy since 2002, currently allowing two children per family, on obstetric workload in a regional referral center in China.

**Methods:**

In a retrospective cohort study, temporal changes were examined in relation with maternal demographics, incidence of women with high risk pregnancies and resource statistics in our hospital in managing singleton viable pregnancies (birth from 28 weeks gestational age onwards) for the period 2005–2017.

**Results:**

During this 13-year period, the number of singleton livebirths from 28 weeks gestational age onwards was 49,479. Annual numbers of births increased from 1,941 to 2005 to 5,777 in 2017. There were concomitant and significant increases in the incidence of multiparous women (10.6–50.8 %), of age ≥35 years (6.5–24.3 %), with prior caesarean Sec. (2.6–23.6 %), with ≥3 previous pregnancy terminations (1.0–4.9 %), with pre-gestational diabetes (0.2–0.9 %), and with chronic hypertension (0.2–1.2 %). There were associated increases in beds and staff complement and reduced average hospital stay. Nevertheless, while the workload of medical staff remained stable with increasing staff complement, that of midwives increased significantly as reflected by the total births: midwife ratio which increased from 194.1:1 to 320.9:1 (*p* < 0.001).

**Conclusions:**

In our hospital, progressively increasing numbers of annual births in combination with an increased incidence of women with high risk pregnancies took place following the revised national childbirth policy. Only the increase in medical and nursing, but not midwifery, staff was commensurate with workload. Remedial measures are urgently required before the anticipated progressive increase in care demand would overwhelm maternity care with potentially disastrous consequences.

## Background

Over the past two decades, China has experienced rapid economic growth with increasing prosperity and expanding urbanized areas. Large scale migration from rural areas into cities is the major factor accounting for the enormous population growth in urbanized areas and cities [1–3]. In association with this is a concomitant increase in individuals with underlying diseases and medical risk factors, which is translated into an escalating demand for health care [2, 4–6]. One of the more prominent demands has to do with maternity care, since the majority of the migrant population are young adults of reproductive age, seeking jobs and employment opportunities in the cities. This follows the relaxation of the national one-child policy to allow two children per family since 2002, which has led to not only increasing numbers of births, but also of women with high risk pregnancies and obstetric complications [7–9]. The magnitude of the challenges on existing maternity care and the rate of increasing demands, however, remain unclear in the absence of any studies.

As a non-profit-making public comprehensive hospital supported and regulated by the city government, our hospital is the leading tertiary referral center in Yunnan Province, which is a less affluent province located in south-western China. Over the past 15 years, we have experienced an escalating number of births in association with increasing demands on a wide spectrum of care, including pregnancy dating, prenatal diagnosis, screening and management of obstetric complications. At the same time needs arose for additional hospital beds to accommodate women admitted with obstetric complications, such as preterm labour and antepartum haemorrhage, interventions for births complicated with placenta praevia and accreta due to the high rate of prior caesarean section (CS) and the concomitant requirement for additional beds in the neonatal unit to accommodate newborns with complications needing intensive care. This led to an increasing workload which progressively overwhelmed the available medical and nursing staff. It has become increasingly clear that data on the extent of all these increases must be examined to enable appreciation of current requirements and future projections of resource allocations. As yet, however, no Chinese studies have examined temporal changes, especially in tertiary referral centers which provide back up for all other hospitals.

We therefore performed a retrospective review of maternity care in our hospital from 2005 to 2017 in order to elucidate temporal changes in maternal demographics, incidence of risk factors and birth-related statistics, in order to assess the burden of increasing workload on current resources. This could provide the basis for additional resources required to cater for future demands of maternity care.

## Methods

### Subjects and Setting

This was a retrospective cohort study utilizing data from the hospital statistics and birth register of the First Affiliated Hospital of Kunming Medical University, a tertiary referral hospital in Yunnan Province in south-western China, from January 2005 to December 2017. During the study period, 51,941 pregnant women with complete data were collected in the database. As the greatest and most consistent increase existed in singleton pregnancies with livebirths, we excluded 1,042 births (2.0 %) ending either in stillbirth at any gestational week or birth below 28 weeks gestational age (in China, viable fetus is defined as those born after 28 gestational age) and pregnancies terminated for fetal aneuploidy or major defects. Reasons for exclusion were that the number of these births was small, and although more related counselling work was needed for them, clinical management resources such as intrapartum fetal monitoring and neonatal management and nursing care were not generally required. We also excluded 1,420 (2.7 %) multifetal pregnancies for the sake of a more comparable baseline, because they often included more severe maternal and fetal complications that would require more clinical management and medical resources than singleton pregnancies. For the final analysis, we included 49,479 singleton pregnancies with livebirths with gestational age above 28 weeks.

Antenatal care usually starts at 8–14 weeks gestational age, when maternal demographic data are recorded, including parity, age and advanced age (age ≥35 years at birth), educational level (dichotomized as tertiary or below), body mass index (BMI, calculated as weight (kg)/height (m^2^), before pregnancy) [10, 11]. BMI was categorized as either normal (< 25 kg/m^2^) or high BMI (≥ 25 kg/m^2^) based on the WHO recommendations for Asian populations [12]. Significant medical conditions (including chronic hypertension before pregnancy and pre-gestational diabetes mellitus) and past obstetric history (including prior CS and repeated therapeutic terminations of pregnancy (TOP ≥ 3)), and obstetric complications were also captured. Assisted reproductive technology (ART) referred to intrauterine insemination, in vitro fertilization and embryo transfer, and intracytoplasmic sperm injection, and only singleton pregnancies were included in the analysis. Gestational age was determined based on either the last menstrual period or ultrasound examination at 11–14 weeks. Obstetric management is based on clinical indications and in-house protocols established on the basis of medical consensus and literature.

Similar to most public hospitals in China, pregnant women attending the antenatal clinic and admitted for birth in our center are managed by different doctors and nursing staff within the department assigned to specific duties or tasks on rotation. Therefore, a woman would probably see a different healthcare provider at each visit, but the same group of providers would be seen in the course of the pregnancy. In the last two years we are in the process of setting up a special appointment service for women with high risk pregnancies. Our center has implemented a program to control the rate of CS for the past 13 years owing to the increasing popularity of CS on maternal request in China [13]. This is overburdening obstetric care and creates potential hazards for both women and health care providers. This is the background against which our workload and staffing situation are examined.

### Parameters analyzed

We examined numbers and rates of total births, labour induction, CS, admission into the intensive care unit (ICU) for the mother and neonatal intensive care unit (NICU) for the infant, bed occupancy in the maternity ward and average maternal hospital stays, as parameters of workload. The total number of in-patients for bed occupancy estimation included women giving birth, hospitalized pregnant women receiving treatment for complications, TOP due to fetal malformation or maternal complications, and women after childbirth readmitted for puerperal problems. The incidence of women who were multiparous, age ≥ 35 years at birth, high BMI, singleton pregnancies conceived through ART, history of CS and ≥3 TOP, pre-gestational diabetes, chronic hypertension and preterm birth (birth at 28–36^+ 6^ weeks) were used as parameters of high risk groups; and tertiary education as reflection of socio-economic class. Parameters of available resources included total numbers of officially allocated beds in the maternity ward, and the annual number of available healthcare providers. The number of hospitalized in-patients often exceeded that of the officially allocated beds in obstetric wards, so “additional beds” were needed. Bed occupancy rate in the maternity wards was defined as the number of inpatients present at midnight divided by the total number of officially allocated beds in the maternity wards in a given calendar year, which represented the actual utilization of beds in one year [14]. When installation of additional beds was required, the official bed occupancy rate in that year would have exceeded 100 %.

Health care providers in the obstetric department were categorized as obstetricians, nurses who only worked in the ward, and midwives who were involved in labour management and childbirth. Obstetricians are doctors providing all maternity care, except obstetric sonography which for administrative reasons was provided by medical staff from the Department of Medical Imaging. Nursing staff included nurses and midwives, the allocation of whom was based on the nursing administrative system in China. Nurses in the ward are those who provide nursing care in the maternity wards, and are in charge of all nursing care for women and their babies outside the labour ward and operation room. Midwives are nurses who have been trained and qualified in midwifery, working only in the labour ward and are responsible for intrapartum monitoring and childbirth, as well as caring for the mothers and their newborn infants up to two hours postpartum. Obstetric sonography is performed by physicians from the Medical Imaging Department, who received subspecialty training in fetal ultrasonography and were not involved in any other obstetric clinical duties. All medical staff in the analysis were appointed as full-time staff, there being no part-time staff or shared staff, and all statistics were based on the actual number of medical staff on duty in a given year (i.e. excluding those on sick leave, advanced training at other institutions, etc.)

As in most hospitals in China, our care model is one of coexisting midwifery-led and obstetrician-led care. All pregnant women were seen and evaluated by an obstetrician after admission and were categorized into either high-risk or low-risk group based on the condition of the mother and fetus. The midwifery-led care model is mainly catering for women with low risk pregnancies (e.g. full-term cephalic-presenting vaginal birth, uncomplicated pregnancy, spontaneous onset of labour, etc.), while obstetrician-led care is catering for women with high risk pregnancies (preterm birth, multiple pregnancy, complicated pregnancy, induction of labour (IOL), CS and instrumental vaginal birth, etc.). We used the birth: staff ratio, which was defined as the ratio of the total number of births in a particular year to the actual number of medical/nursing staff available in order to assess the workload of medical/nursing staff in the obstetric department. This ratio would reflect the number of births served by a maternity care provider in a given year, irrespective of seniority or rank [15]. According to the standardized training system for resident doctors and nursing staff in China, all newly recruited staff would be rotated in each department of the hospital during the training period of 2–3 years, and each is counted as one staff member regardless of experience.

### Statistical analysis

Maternal demographics were analyzed with χ^2^ test, and Spearman’s correlation was used to assess temporal trends. Statistical software package SPSS Statistics 22.0 (SPSS Inc., Chicago, IL, USA) was used for data analysis, and GraphPad Prism 6.0 Software (GraphPad Software, Inc., SanDiego, CA) for graphics. *P*-values < 0.05 (two-tailed) were regarded as statistically significant.

## Results

**Fig. 1:**
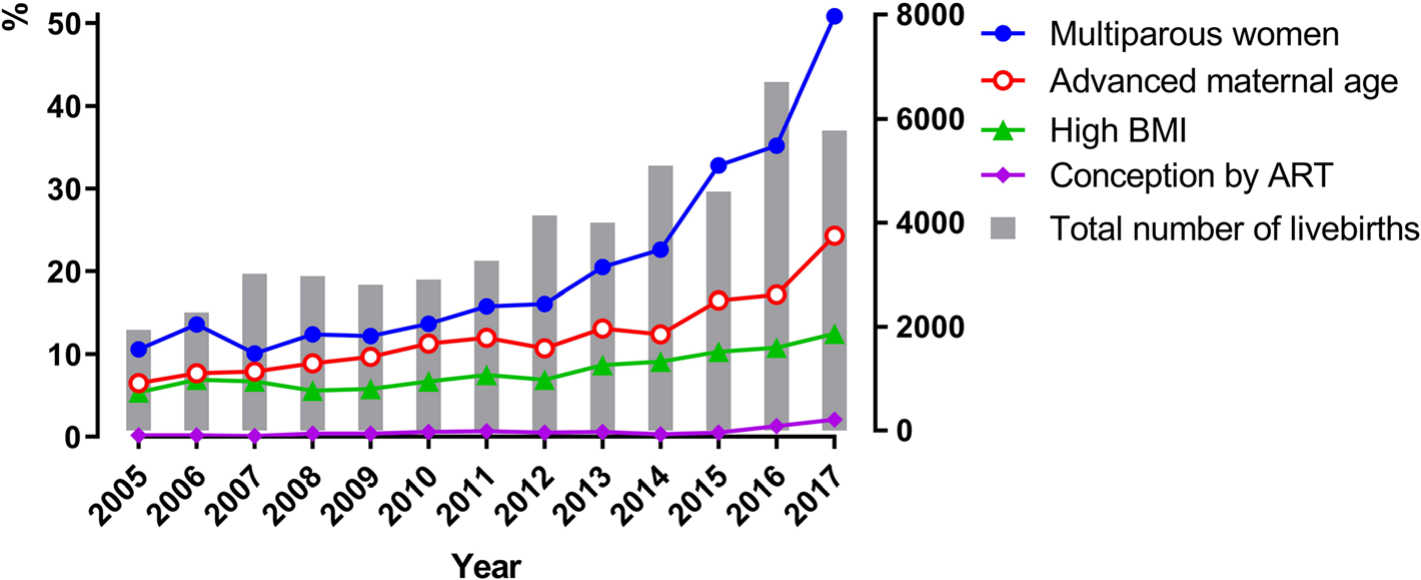
Temporal changes in maternal demographic factors 2005–2017. () Multiparous women; () Advanced maternal age;() High BMI; () Conception by ART; () Total number of livebirths

The total number of singleton livebirths with gestational age of ≥ 28 weeks during these 13 years was 49,479. There was a progressive and significant increase (*p* < 0.001) from 1,941 to 2005 to 5,777 in 2017, peaking in 2016 with 6,700 births (Fig. 1; Table 1). A statistically significant, concomitant increase also occurred in the incidence of multiparous women from 10.6 to 50.8 % (4.8 fold), age ≥ 35 from 6.5 to 24.3 % (4.5 fold) and high BMI from 5.4 to 12.5 % (2.3 fold). There was a statistically significant increase in women conceived by ART from 0.2 to 2.1 % (10.5 fold), the increase being largely related to a big jump in singleton pregnancies conceived through ART since 2016. There was also a statistically significant improvement in socio-economic class with the incidence of tertiary-educated pregnant women increasing from 22.9 to 78.2 % (3.4 fold).

**Table 1:** Changes in Maternal demographics from 2005–2017

Year	Total births (*n* = 49,479)	Multiparas (%)	Age ≥ 35 years (%)	High BMI^a^ (%)	ART pregnancy (%)	Tertiary education^b ^(%)
2005	1941	10.6	6.5	5.4(78/1453)	0.2	22.9(443/1932)
2006	2278	13.6	7.7	6.9(121/1751)	0.2	43.7(996/2278)
2007	3011	10.1	7.9	6.7(173/2565)	0.1	40.5(1209/2983)
2008	2968	12.4	8.9	5.6(151/2687)	0.4	37.2(1102/2960)
2009	2806	12.2	9.7	5.8(161/2781)	0.4	40.1(1123/2799)
2010	2900	13.7	11.3	6.7(193/2884)	0.6	43.9(1268/2891)
2011	3267	15.8	12.0	7.5(244/3246)	0.7	45.3(1481/3267)
2012	4140	16.1	10.7	6.9(286/4118)	0.5	48.4(2005/4140)
2013	4000	20.5	13.1	8.7(347/3983)	0.6	49.8(1991/4000)
2014	5096	22.6	12.4	9.1(463/5085)	0.3	55.5(2829/5094)
2015	4595	32.8	16.5	10.3(473/4578)	0.5	57.1(2617/4581)
2016	6700	35.2	17.2	10.8(721/6700)	1.3	61.0(4085/6700)
2017	5777	50.8	24.3	12.5(721/5749)	2.10	78.2(4516/5777)
Difference	*P <* 0.001	*P* < 0.001	*P* < 0.001	*P* < 0.001	*P* < 0.001	*P* < 0.001
Correlation	*P <* 0.001	*P* < 0.001	*P* < 0.001	*P* < 0.001	*P* < 0.001	*P* < 0.001

Results expressed in % unless specified, comparison of difference by χ^2^ test, correlation analysis by Spearman’s test; *BMI* body mass index, *ART* assisted reproduction technology

^a^Missing = 1899; ^b^Missing = 77

When examining high risk groups, there was a 9.1 fold increase in incidence of prior CS from 2.6 to 23.6 % and that of previous TOP ≥3 by 4.9 fold from 1.0 to 4.9 %. Incidence of pregnant women with pre-gestational diabetes increased from 0.2 to 0.9 %, and chronic hypertension from 0.2 to 1.2 % (6 fold, Table 2). Incidence of preterm births increased 1.9 fold from 5.0 to 9.3 %, and labour induction 12.3 fold from 1.4 to 17.2 % (both statistically significant). On the other hand, CS incidence fell from 42.1 to 30.8 % (*p* < 0.001). There was no increasing trend for maternal ICU admission, while NICU admission increased statistically significant by 1.6 fold from 13.8 to 21.8 % (Table 2).

**Table 2:** Changes in risk factors from 2005 to 2017

Year	Prior CS	Previous TOP ≥ 3	Pre-gestational diabetes	Chronic hypertension	Preterm birth	Labour induction	Total CS rate	ICU admission	NICU admission
	(%)	(%)	(%)	(%)	(%)	(%)	(%)	(%)	(%)
2005	2.6	1.0	0.2	0.2	5.0	1.4	42.1	0.6	13.8
2006	4.4	1.2	0.4	0.1	5.9	1.2	39.0	0.6	11.4
2007	3.9	1.0	0.4	0.1	6.6	1.9	39.0	0.5	10.1
2008	3.7	0.8	0.5	0.2	6.6	2.9	39.6	0.5	14.5
2009	5.0	0.7	0.4	0.5	7.9	3.0	41.9	0.3	16.1
2010	6.1	0.9	0.3	0.8	8.3	2.4	45.2	0.5	22.1
2011	6.7	0.6	0.4	0.7	8.3	2.0	35.4	1.0	19.5
2012	7.6	0.2	0.6	0.7	7.8	13.5	32.7	0.8	12.4
2013	9.6	0.1	0.8	1.0	8.1	23.0	36.2	0.9	14.7
2014	10.7	0.8	0.6	1.0	7.7	19.6	33.2	0.8	15.5
2015	15.6	0.3	0.8	1.2	8.3	24.5	32.9	0.8	19.7
2016	17.8	3.1	0.7	1.0	8.2	14.9	25.8	0.6	22.3
2017	23.6	4.9	0.9	1.3	9.3	17.2	30.8	0.3	21.8
Difference	*P <* 0.001	*P <* 0.001	*P <* 0.001	*P <* 0.001	*P* < 0.001	*P* < 0.001	*P* < 0.001	*P* < 0.001	*P* < 0.001
Correlation	*P <* 0.001	*P <* 0.001	*P <* 0.001	*P <* 0.001	*P* < 0.001	*P* < 0.001	*P* < 0.001	*P =* 0.605	*P* < 0.001

Results expressed in N (%), comparison of difference by χ2 test, correlation analysis by Spearman’s test; *CS* caesarean section, *TOP* therapeutic termination of pregnancy

Our hospital has been expanded during these 13 years with officially allocated maternity beds increasing from 40 to 72 (1.8 fold increase), but the total number of in-patients with singleton pregnancies increased 2.7 fold from 2757 to 7340 (Table 3). Average bed occupancy rate, however, showed a slight but non-significant decrease from 133.8 to 107.2 %, attributable to the significantly reduced average hospital stay from 7.2 to 3.9 days, a 45.8 % reduction. In association with increased workload, statistically significant increases occurred in staff complements of all categories (Table 4). Nevertheless, while the number of obstetricians increased by 4 fold, and nurses in the ward increased by 3.8 fold, it was only 1.8 fold for midwives. The dissimilar increase in different staff categories explained the different patterns and changes in workload of medical and nursing staff. The total births: obstetrician ratio showed a slight improvement from 277.3:1 to 206.3:1, and the pattern was similar for nursing staff in the ward which improved slightly from 129.4:1 to 101.4:1. On the other hand, the total births: midwife ratio deteriorated as it increased markedly from 194.1:1 to 320.9:1 towards the end of the study period (Table 4).

**Table 3:** Bed usage in the maternity wards from 2005 to 2017

Year	Total inpatients (N)	Official allocated beds (N)	Bed occupancy rate (%)	Average hospital stay (day)
2005	2757	40	133.8	7.2
2006	3263	40	128.1	5.7
2007	3566	40	150.4	6.2
2008	3503	40	165.3	7.0
2009	3370	40	134.5	5.9
2010	3718	40	135.3	5.3
2011	3891	46	150.6	6.1
2012	4629	61	99.6	4.6
2013	4699	67	95.6	4.9
2014	5880	67	148.5	6.3
2015	5697	70	122.0	5.5
2016	7772	72	156.5	5.3
2017	7340	72	107.2	3.9
Correlation	*P<*0.001	*P<*0.001	*P =* 0.505	*P =* 0.019

Correlation analysis by Spearman’s test

**Table 4:** Changes of manpower and workload from 2005 to 2017

Year	Total births	Obstetricians (N)	Nurses in the ward (N)	Midwives (N)	Total births: Obstetrician ratio	Total births: Nurse in the ward ratio	Total births: Midwife ratio
2005	1941	7	15	10	277.3:1	129.4:1	194.1:1
2006	2278	8	17	10	284.8:1	134.0:1	227.8:1
2007	3011	8	16	13	376.4:1	188.2:1	231.6:1
2008	2968	9	15	14	329.8:1	197.9:1	212.0:1
2009	2806	10	18	14	280.6:1	155.9:1	200.4:1
2010	2900	12	20	14	241.7:1	145.0:1	207.1:1
2011	3267	14	21	14	233.4:1	155.6:1	233.4:1
2012	4140	16	27	16	258.8:1	153.3:1	258.8:1
2013	4000	18	27	17	222.2:1	148.1:1	235.3:1
2014	5096	20	32	18	254.8:1	159.3:1	283.1:1
2015	4595	24	46	18	191.5:1	99.9:1	255.3:1
2016	6700	28	57	15	239.3:1	117.5:1	446.7:1
2017	5777	28	57	18	206.3:1	101.4:1	320.9:1
Correlation	*P <* 0.001	*P <* 0.001	*P <* 0.001	*P <* 0.001	*P* = 0.001	*P* = 0.156	*P* < 0.001

Correlation analysis by Spearman’s test

## Discussion

As a standard tertiary referral center in southwestern China, our hospital has experienced an enormous increase not only in overall workload but also in the proportion of women with high risk pregnancies within the obstetric population from 2005 to 2017. These increases reflected both increasing referrals from other regional hospitals, but more importantly a disproportional increase in the number of women with significant risks such as advanced maternal age, prior CS, TOP ≥3 times, pre-gestational diabetes and chronic hypertension. While the annual number of inpatients correlated with the number of births, these increases exceeded the increase in maternity beds, which only meant that the average hospital stay had to be reduced, from 7.2 days in 2005 to 3.9 days in 2017. Such a phenomenon could only be explained by progressive changes in practice, reflected in increasing incidence of labour induction for high risk pregnancy and decreasing incidence of CS, the latter probably explaining the decreasing bed occupancy rate as well. Although these changes were associated with increasing staff complements, especially for obstetricians and nurses in the ward, midwives had to care for more women during childbirth. Taken together, these statistics would suggest a progressive increase in the efficiency of the department as a whole, a reflection of streamlining and improving provided care as well as standards of care, as increases in NICU admission were not accompanied by a similar increase in ICU admission.

There has been considerable evidence suggesting that the safety of the childbearing women and newborns is closely related to the quality of midwifery care [16, 17]. Safe workload for midwives is a fundamental issue, and lower caseloads are required where women and their babies are more likely to experience risk factors in pregnancy [18]. At the same time, rapid annual increases of both incidence and actual numbers of women with high risk pregnancies, who would require obstetrician-led care instead, could very likely overwhelm obstetric and nursing staff’s ability to cope. The increase in obstetricians was provided by new doctors who were mostly young and inexperienced obstetrician-trainees, who would require time to acquire the skills in handling the disproportionate increase of complicated pregnancies. Therefore although staff complement would be regarded as sufficient on paper, dilution of experienced staff would create a situation similar to staff shortages.

As obstetrics involves the management and care of both mothers and babies, it is important to develop specific guidelines or standards for obstetric staffing. The Royal College of Obstetricians & Gynaecologists (RCOG) and Royal College of Midwives (RCM) suggested that labouring women should receive 1:1 individual care by midwives and for women with high risk pregnancies the midwife-to-woman ratio should be up to 1.2–1.4:1 and recommended that the minimum midwife-to-woman ratio should be 1:28 for safe level of care to ensure the capacity to achieve one-to-one care in labour [19]. Queensland Nurses Union has developed a standard of minimum requirements for safe staffing in midwifery care [17, 18, 20]. Nurse/midwives-to-woman ratio should be no less than 1:1 for management of active labour and 1:4 − 1:6 for inpatient management, while newborns must count in staffing calculations in postnatal wards. An observational study about the association between midwifery staffing and outcomes in maternity care in England reported obstetric staff-birth ratios in over 100 hospitals with average numbers of birth per obstetrician registrar of 327.7 (range from 56 to 1133) and 257.6 (range from 26 to 5070) for registered nurses, while the number of births per midwife was 31.5 (range from 9 to 81) [15]. When comparing with our data, births per obstetrician and nurses in the ward was similar to these international data, while births per midwife in our centre was remarkably greater than in that study. In our center, births per midwife were around 200 per year, peaking at over 400 in 2016, when the childbirth policy changed (Table 4), significantly higher than the RCOG recommended midwife-to-woman ratio of 1:28. Although this might be partly explained by the different maternity systems and working models of medical staff in different countries, it still remained a major concern about safe childbirth, since in practice it was indeed very difficult to provide one-to-one individual care for women in active labour in most public hospitals in China. Therefore an urgent need exists for an obstetric staffing guideline or recommendations to cater for the maternity system and native conditions in China to ensure safe childbirth.

An US study on physicians’ workload that offered a minimal potential for error or harm, has found that 40 % of physicians reported a typical episode of inpatient care which had fallen below a safe level occurring at least monthly, with a frequency of greater than once per week in 36 %, indicating that > 20 % of physicians reported that their average workload would likely contribute to patient morbidity and even mortality [21]. A ‘busy day’ effect on perinatal complications has been demonstrated, especially during weekends [22]. A survey in nine western countries found an association between nurse staffing shortages and missed care, related with 30-day case-mix adjusted mortality [23]. In France, combination of excessive nursing workload and inexperienced medical staff was associated with seasonal peaks in severe adverse events in the adult ICU [24]. With a potential of obstetric complications in every birth, even among low-risk pregnancies, the set-up of a high dependency unit in the birthing suite, the need to perform sequential and occasionally concurrent surgical procedures in the obstetric operation theatre, the fact that every birth involves at least two patients – mother and infant - and the fact that there is no control over when and how many pregnant women would be admitted requiring immediate attention, would render a busy tertiary obstetric referral center an even more hazardous place than the ICU. Especially in low-income countries, staff shortages and workload are key factors, together with inadequate obstetric skills, that would demoralize obstetric health care providers, a significant proportion of whom considering to leave their current posts [25]. Thus an imbalance between workload and staffing has implications beyond a mere extension of working hours for health care providers.

In our study, we managed to reduce the extremely high CS-rate significantly to a still high overall CS-rate without any concomitant increase in the ICU admission rate despite the rapidly escalating workload. While this could be attributed to a well-organized care system with sound and proven management protocols, under close supervision by experienced obstetric staff, this must not be taken for granted and there is no room for complacency. The current national two-child policy, officially implemented since October 2015, will undoubtedly lead to increasing demand and proportions of women with high risk pregnancies, one reason being the high CS-rate.

It is always prudent to prevent obstetric care from reaching the turning point which could erode standards of maternity care with increasing adverse outcomes for women and newborns. It is thus imperative to instigate remedial measures as soon as possible. Related guidelines for medical care provision in the maternity system, such as the UK RCOG-guideline in planning, reviewing and auditing nationwide care provision has just been introduced in China [26]. As a local tertiary referral hospital, we suggested some feasible solutions from our perspective. First step would be to invest in staff expansion and training through comprehensive programs organized by national professional bodies, coupled with improvements in facilities and logistical support, such as in-utero transfer of women with high risk pregnancies. Regional networks must be strengthened with enhanced liaison among centers with designated coordinators who are constantly updated of available resources such as NICU beds. At the same time, society must be educated about how maternity care is provided and cost-effective ways of utilizing care, including knowledge on the pros and cons of various medical interventions, their short and long term sequelae and the rationale of decision-making. Last but not least, the health ministry must be timely informed of the current and projected needs so that adequate funding and resources can be allocated in time.

## Strengths and limitations

Strength of our study lies in the fact that as a single-center study, uniformity in diagnosis, data collection and clinical management existed for all women, with regular clinical audit to maintain standards according to international consensus. The significantly declining, albeit still high CS rate is a testimony of the obstetricians’ compliance to our protocols for primary and repeat CS, especially in our attempts since mid-2013 to promote trial of vaginal birth after CS[13, 27].

Limitations of our study were related to its retrospective nature, spanned 13 years during which there have been changes in clinical management. Another limitation is that owing to the nature of a tertiary referral hospital, our patient population could have represented a selected group of higher risk as well as better educated women. Information on the educational level of each woman was based on self-reporting, as we have no means of checking their credentials. The actual educational level attained under the label of tertiary education could not be sub-categorized further, but given the rigorous execution of the national education policy, the percentage of women without any formal education could only be very small (0.4 %). Also incomplete data existed regarding sociodemographic characteristics due to difficulties in obtaining or validating data such as past obstetric and medical history, which might also affect assessment for workload [28]. These included proportions of emergency admissions and transfers, admissions during out of office hours and for those without regular antenatal care, different skills of different medical staff, changes of the hospital information system and workflow, actual working hours (overtime hours by medical staff are usually disregarded in China), all leading to difficulties to accurately evaluate workload. Finally, despite empirical evidence of improved efficiency and standards of care, a proper audit would be necessary to confirm these impressions [29].

## Conclusions

A picture of rapid increase of obstetric workload in a tertiary referral center in a less affluent part of China could be documented over recent years. Increases in the total number of births, women with high risk pregnancies and enlarging discrepancies between the numbers of health care providers, especially midwives, and escalating workload, all create enormous challenges for the provision of maternity care in the coming decade. Countermeasures must be designed and implemented without delay in order to ensure safe childbirth for our women and their offspring.

## Data Availability

Datasets are not publicly available due to the fact that it contains personal information, but are available as de-identified format from the corresponding author on reasonable request.

## Abbreviations

ART: Assisted reproductive technology
BMI: Body mass index
CS: Caesarean section
ICU: Intensive care unit
NICU: Neonatal intensive care unit
TOP: Termination of pregnancy
